# Altered Mechanobiology of PDAC Cells with Acquired Chemoresistance to Gemcitabine and Paclitaxel

**DOI:** 10.3390/cancers16223863

**Published:** 2024-11-18

**Authors:** Alessandro Gregori, Cecilia Bergonzini, Mjriam Capula, Rick Rodrigues de Mercado, Erik H. J. Danen, Elisa Giovannetti, Thomas Schmidt

**Affiliations:** 1 Cancer Biology and Immunology, Cancer Center Amsterdam, 1081 HV Amsterdam, The Netherlands; a.gregori@amsterdamumc.nl (A.G.); elisa.giovannetti@gmail.com (E.G.); 2 Department of Medical Oncology, Amsterdam UMC, Location Vrije Universiteit Amsterdam, 1081 HV Amsterdam, The Netherlands; 3 Physics of Life Processes, Huygens-Kamerlingh Onnes Laboratory, Leiden University, 2333 CA Leiden, The Netherlands; rodrigues@physics.leidenuniv.nl; 4 Leiden Academic Center for Drug Research, Leiden University, 2333 CC Leiden, The Netherlandse.danen@lacdr.leidenuniv.nl (E.H.J.D.); 5 Fondazione Pisana per La Scienza, 56017 San Giuliano Terme, Italy

**Keywords:** PDAC, mechanobiology, chemoresistance

## Abstract

**Background**: Pancreatic ductal adenocarcinoma acquired resistance to chemotherapy poses a major limitation to patient survival. Despite understanding some biological mechanisms of chemoresistance, much about those mechanisms remains to be uncovered. Mechanobiology, which studies the physical properties of cells, holds promise as a potential target for addressing the challenges of chemoresistance in PDAC. Therefore, we, here in an initial step, assessed the altered mechanobiology of PDAC cells with acquired chemoresistance to gemcitabine and paclitaxel. **Methods**: Five PDAC cell lines and six stably resistant subclones were assessed for force generation on elastic micropillar arrays. Those measurements of mechanical phenotype were complemented by single-cell motility and invasion in 3D collagen-based matrix assays. Further, the nuclear translocation of Yes-associated protein (YAP), as a measure of active mechanical status, was compared, and biomarkers of the epithelial-to-mesenchymal transition (EMT) were evaluated using RT-qPCR. **Results**: The PDAC cells with acquired chemoresistance exert higher traction forces than their parental/wild-type (WT) cells. In 2D, single-cell motility was altered for all the chemoresistant cells, with a cell-type specific pattern. In 3D, the spheroids of the chemoresistant PDAC cells were able to invade the matrix and remodel collagen more than their WT clones. However, YAP nuclear translocation and EMT were not significantly altered in relation to changes in other physical parameters. **Conclusions**: This is the first study to investigate and report on the altered mechanobiological features of PDAC cells that have acquired chemoresistance. A better understanding of mechanical features could help in identifying future targets to overcome chemoresistance in PDAC.

## Simple Summary

Pancreatic ductal adenocarcinoma (PDAC) aggressive and chemoresistant phenotype poses a major challenge to patient treatment. The study of the mechanical features of chemoresistant cells could offer new strategies for therapy development. Therefore, in this study, we investigated how chemoresistant PDAC cells generate forces, characterized their movement in 2D and in 3D, and finally studied the expression of EMT markers and the localization of YAP in these cells. We found that chemoresistant PDAC cells apply higher traction forces than their parental clones on micropillar arrays and their single-cell motility is altered. However, we did not find a pattern either in the alteration of 3D migration, which was rather cell line-dependent, or in EMT markers expression and YAP localization. This study paves the way for a deeper characterization of mechanobiology in chemoresistant PDAC cells and highlights the challenges connected with the use of different models.

## 1. Introduction

Pancreatic ductal adenocarcinoma (PDAC) is the most common type of pancreatic cancer and is associated with a dismal prognosis. The 5-year survival is reached by only 13% of all the patients (see 2024 update at Pancreatic Cancer Five-Year Survival Rate Increases to 13%—Pancreatic Cancer Action Network (pancan.org)) [1]. The lack of early diagnosis, limited therapeutic options, and inherent or acquired chemoresistance all concur to the poor prognosis [2]. Besides surgical resection, for which a small portion of patients is eligible, chemotherapy is the only available treatment. Currently, the combination regimens FOLFIRINOX or gemcitabine plus nab-paclitaxel are being employed to treat patients with inoperable PDAC [3]. However, in the majority of the patients, rapid chemoresistance occurs, with the underlying biological mechanisms remaining largely elusive. Therefore, mechanobiology, which analyzes physical forces in a biological context, is gaining attention in the studies on the chemoresistance of PDAC [4].

Cellular mechano-transduction, the process by which mechanical cues are converted into intracellular molecular signals, along with interactions with the surrounding extracellular matrix (ECM), plays a pivotal role in cell homeostasis and signaling. Increasing evidence shows that this phenomenon is particularly critical in cancers that develop abnormal ECM, causing tumor progression, increased metastatic potential, and drug resistance [5,6,7]. PDAC is characterized by a drastic change in the ECM, reflected by tissue stiffening. Indeed, PDAC progression is accompanied by a desmoplastic reaction, which produces a dense stroma constituting up to 90% of the tumor volume, and increases tissue stiffness up to 50 kPa in terms of Young’s modulus [8]. Therefore, PDAC cells are subject to many mechanical stimuli which are thought to promote tumor progression and therapy resistance. One such mechanism that we previously reported is the overexpression of the integrin alpha 2 (ITGA2), a cellular stiffness sensor that promotes gemcitabine resistance in PDAC [9]. Additionally, Rice and collaborators showed the involvement of epithelial-to-mesenchymal transition (EMT) in inducing the chemoresistance of PDAC cells [10].

Metastatic dissemination and cell migration are widely affected by stromal stiffness in many cancers [11]. In general, cells generate forces to migrate through the ECM and a correlation between metastatic potential and increased force generation has been previously described for many types of cancers [12]. Likewise, it is believed that EMT is required for cells to lose ECM adhesion and migrate [13]. EMT has been investigated in PDAC in relation to metastasis and inherent or acquired chemoresistance [10,13,14,15]. The induction of EMT and activation of cellular forces are translated into mechanical stimuli, which in turn trigger Yes-associated protein (YAP) nuclear translocation and downstream signaling [10]. Moreover, YAP signaling was linked to chemoresistance to *KRAS* G12C inhibitors in non-small cell lung cancer [16,17]. Interestingly, *KRAS* is often mutated in PDAC, and its activity appears to depend on the tissue and the allele where the mutation occurs [18].

Despite those general observations, there is limited understanding regarding whether cells with acquired chemoresistance exhibit a deregulated mechano-transduction. We hypothesized that exposing PDAC cells to paclitaxel, a drug that inhibits microtubule disassembly, impacts cytoskeleton activity and/or mechanical signaling. Consequently, we here investigated and report, for the first time, that PDAC cells with acquired chemoresistance to gemcitabine and paclitaxel display an altered mechanical phenotype. For investigation, the present study assessed force generation in conditions of varying stiffness in five PDAC cell lines, and in six chemoresistant subclones. Additionally, single-cell motility in 2D and 3D collagen-embedded spheroids was investigated to determine the invasive potential. Finally, Hippo signaling and the EMT-mediators of mechano-transduction were analyzed, revealing that mechanical forces in chemoresistant cells were not accompanied by YAP nuclear translocation and changes in the expression of EMT genes.

## 2. Methods

### 2.1. Cell Culture

One non-tumor cell line, HPDE, kindly provided by Ming Tsao (Ontario Cancer Institute, Toronto, ON, Canada), and five PDAC cell lines were employed in this study: CAPAN-1 (epithelial phenotype) and BxPC-3 (epithelial phenotype) were purchased from the American Type Culture Collection (ATCC, Manassas, VA, USA), PATU-T (mesenchymal phenotype) were kindly provided by Dr. Irma van Die (Amsterdam UMC, Amsterdam, The Netherlands), and SUIT-2.028 and SUIT-2.007 (epithelial and mesenchymal phenotype, respectively) were kindly provided by Dr. Adam Frampton (Imperial College London, London, UK). All the gemcitabine-resistant and paclitaxel-resistant cells were generated by continuous drug exposure over a period of 12 months, as described in detail by Bergonzini and colleagues [19]. Briefly, at the end of the procedure, the IC50s of the resistant cells were more than 100-fold higher compared to the respective parentals, as confirmed by proliferation assays. HPDE were cultured in KGM medium (Lonza, Basel, Switzerland), PATU-T in DMEM (Thermo Fisher Scientific, Waltham, MA, USA), and all the other cell lines in RPMI medium (Thermo Fisher Scientific, Waltham, MA, USA). All the cells were supplemented with 10% heat-inactivated new-born calf serum (Biowest, Nuaillé, France) and 1% penicillin/streptomycin (Lonza, Basel, Switzerland) and kept at 37 °C and 5% CO_2_. The cells were periodically tested for mycoplasma contamination.

### 2.2. Elastic Micropillar Arrays

In this study, we used elastic micropillar arrays (μPAs) of polydimethylsiloxane (PDMS, Sylgard 184, Dow Corning, Midland, Michigan, USA) arranged in hexagonal geometry, of 2 μm diameter, 4 μm spacing, and a height (effective Young’s modulus) of 3.2 μm (142 kPa), 4.1 μm (47 kPa), 6.1 μm (29 kPa), and 6.9 μm (11 kPa), respectively. μPAs were generated as previously described in detail [20,21]. Briefly, a 1:10 PDMS mixture (crosslinker/base ratio) was poured into a negative mold made in silicon wafers and cured for 20 h at 110 °C. Next, μPA were peeled off the wafers, activated with ultraviolet light for 10 min, and coated with fibronectin (F1141; Sigma-Aldrich, Saint Louis, MO, USA) using micro-contact printing. A mixture of 1:5 AlexaFluor488-labeled to unlabeled fibronectin was used for coating the top of the pillar arrays.

### 2.3. Cell Seeding, Immunostaining, and Microscopy

The cells were seeded on the μPAs and incubated at 37 °C for 16–19 h in order to allow for attachment and spreading, but to prevent duplication. Subsequently, the cells were fixed for 15 min in 4% paraformaldehyde (PFA), permeabilized for 10 min in 0.1% Triton X-100 (Sigma-Aldrich, Saint Louis, MO, USA), blocked for 1 h in 5% BSA (Sigma-Aldrich, Saint Louis, MO, USA), and stained for 1 h with AlexaFluor568-Phalloidin (Invitrogen, Thermo Fisher Scientific, Waltham, MA, USA). Finally, nuclei were counterstained with DAPI (Invitrogen, P36966, Waltham, MA, USA). The μPAs were then flipped upside-down on a 25 mm diameter #1.5 coverglass, and imaged using a 100× oil-immersion objective on an Axiovert200 optical microscope (Carl Zeiss, Oberkochen, Germany) equipped with a spinning disk unit (CSU-X1; Yokogawa Electric, Musashino, Tokyo, Japan), and an emCCD camera (iXon 897; Andor Labs, Morrisville, NC, USA).

### 2.4. Pillar Deflection Analysis

Pillar deflection was analyzed using a custom-designed Matlab (Matlab R2018a; MathWorks, Natick, MA, USA) script as previously described [20]. Briefly, the center of each pillar was determined to a precision of ~50 nm. Pillar displacements from the original positions within a hexagonal grid were used to map the force-induced pillar deflections (Δ*x*). The force was calculated from the deflection and the pillar’s elastic constant (*k*) using Hooke’s law: F=k∗Δx.

Traction forces were defined as the inward-pointing forces (F*_in_*). All the outward-pointing forces were excluded from further analyses.

### 2.5. YAP Nuclear Translocation

The cells were seeded on the μPAs and immunostained as described above. The cells were incubated for 1 h with a Yes-associated protein (YAP) primary antibody (Santa Cruz Biotechnology, Dallas, TX, USA, sc101199, 1:200) followed by secondary antibody labeling. The cells were then imaged on a Nikon TEi2 confocal microscope equipped with an automated stage, and controlled by the NIS Element software AR 5.11.03 (Nikon Instruments Inc., Melville, NY, USA). YAP nuclear translocation was calculated as the percentage of nuclear YAP over the total YAP:

$$\frac{{YAP}_{nuc}-{YAP}_{bkg}}{\;\left({YAP}_{nuc}-{YAP}_{bkg}\right)+\left({YAP}_{cyt}-{YAP}_{bkg}\right)\;}$$

where *YAP_nuc_* is nuclear YAP, *YAP_bkg_* is the background in the YAP channel outside the cell area, and *YAP_cyt_* is cytosolic YAP. The correlation between YAP and mean force per pillar was calculated in R Studio (v. 2024.04.2–R v. 4.4.1) using the linear regression model of the *ggplot* package.

### 2.6. Quantitative PCR (RT-qPCR)

The cells were seeded in 6-well plates (VWR, Radnor, PA, USA). RNA was extracted from adherent cells 48 h post-seeding using TriZol Reagent (Invitrogen, Thermo Fisher Scientific, Waltham, MA, USA) according to the manufacturer’s instructions. A total of 500 ng of RNA was used to retrotranscribe cDNA with the First Strand cDNA synthesis kit (#K1612, Thermo Fisher Scientific, Waltham, MA, USA) following manufacturer instructions. RT-qPCR was carried out with Sso Advanced Universal SYBR Green Supermix (#172-5271, Bio-Rad, Hercules, CA, USA) in a CFX96 Real-Time System (Bio-Rad, Hercules, CA, USA) apparatus. Primer sequences (Invitrogen, Thermo Fisher Scientific, Waltham, MA, USA) for E-cadherin (*E-cad*), N-cadherin (*N-cad*), Vimentin (*Vim*), and Beta-actin (*β-actin*) can be found in the Supplemental Table S1.

### 2.7. Single-Cell Motility

96-well Screenstar black µClear plates (#655866, Greiner Bio-One GmbH, Alphen aan den Rijn, The Netherlands) were coated with 20 µg/µL rat-tail collagen (Ibidi, Gräfelfing, Germany) or 20 µg/µL human fibronectin (Corning, Corning, NY, USA) in Ultrapure water for 1 h at 37 °C. After removing the coating solution, the coated wells were washed three times with PBS. The PDAC cells were seeded at different densities: PATU-T (6000 cells/well); SUIT-2.007 and SUIT-2.028 (7000 cells/well). After 24 h, all the cell lines were treated with 5 µM Verapamil or medium (control) for 1 h. Verapamil was used to allow Hoechst accumulation in PR cells, which overexpress ABCB1, as previously described [19]. The cells were incubated with Hoechst 33342 (Thermo Fisher Scientific, Waltham, MA, USA) solution for 1 h, then the medium was refreshed. A concentration of 1.25 µg/µL bosutinib (Biosynth, Staad, Switzerland) was used as a positive control for inhibited migration and added to selected wells before imaging. The plates were then imaged with a 20× objective on an ImageXpress micro XLS imager (Molecular Devices, San Jose, CA, USA) equipped with an incubator to maintain the cells at 37 °C and 5% CO_2_. Four fields per well were imaged every 12 min for 16 h. Two wells per condition were analyzed, except for bosutinib which was tested in a single replicate. Data from at least three experiments were collected. The images were analyzed with a Matlab script as previously described [22] after adaptation to identify and localize cell nuclei. The cells were identified and localized from thresholded images. Noise (object size < 1 μm^2^) and large clusters (object size > 9 μm^2^) were discarded from further analysis. From the position data, trajectories were determined and mobility analysis was applied. Cell mobility was analyzed in terms of the change in their mean-squared displacement (*msd*) with lag time (*t_lag_*) between two observations. For the analysis, we assumed both a diffusive motion characterized by a diffusion constant (*D*) and a directed motion characterized by a velocity (*v*) (for further details consider the Supplementary Material). Subsequently, the diffusive fraction of the motility pattern was characterized by a diffusive fraction (*f_D_*), which we defined as the ratio of the diffusive part of the *msd* to the total *msd* at a fixed lag time, *t_D_* = 10 s.

Each trajectory was characterized by those three parameters. From the single-trajectory analysis, the means and standard deviations of the cell populations were subsequently determined as presented in the Results Section. The equations describing mean-squared displacement and diffusive fraction can be found in the Supplementary Material.

### 2.8. 3D ECM Remodeling Ability

PDAC spheroids embedded in 1 mg/mL rat-tail type-I collagen gels were obtained by automated micro-injection as previously described [9,23]. The gels were polymerized in 384-well µclear plates (Greiner Bio-One GmbH, Kremsmünster, Austria) at 37 °C for 1 h, washed with growth medium after polymerization 6 times every 15 min, and 1 time for 1 h, before cell injection. The images of tumor spheroids were acquired using a Nikon Eclipse TEi2 inverted scanning confocal microscope equipped with laser lines 405 nm, 488 nm, 561 nm, and 640 nm; an A1R MP scanner; a Nikon encoded and automated stage; and a temperature- and CO_2_-controlled incubator. The microscope was controlled through the NIS Element Software AR 5.11.03 (Nikon Instruments Inc., Melville, NY, USA). Images were acquired after 24 h with a Plan Apo 20×/0.75 NA objective (Nikon Instruments Inc., Melville, NY, USA). For reflection microscopy, collagen fibers were scanned at 561 nm excitation with a 561-blocking dichroic mirror. All the other wavelengths passed a bandpass filter 400–750 nm. The image-stitching function from the software was used to combine 2 × 2 images. At least 19 z-planes, 15 µm apart, were acquired for each spheroid. To analyze the invasion/migration potential of the PDAC spheroids, the samples were fixed, permeabilized, and stained 48 h post-injection with a solution containing 0.05 µM rhodamine phalloidin (Invitrogen, Thermo Fisher Scientific, Waltham, MA, USA), 2 µg/mL Hoechst 33342 (Thermo Fisher Scientific, Waltham, MA, USA), Triton-X 0.1% (Sigma-Aldrich, Saint Louis, MO, USA), and 2% PFA (Santa Cruz Biotechnology, Dallas, TX, USA) and PBS. After an O/N incubation at 4 °C, the gels were washed 3 times with PBS, and images of the spheroids were captured with a Nikon TEi2 confocal microscope and a 20× objective. To obtain the migration area, the z-projection of the spheroid images was analyzed using an in-house Matlab script. Briefly, the z-stack images of spheroids were projected using the standard deviation in the z-direction of the rhodamine phalloidin channel. The foreground was separated from the background using an adaptive threshold. Morphological features, such as the area, were extracted from the projected foreground of the individual spheroids. Images of day 0 were acquired with a phase-contrast microscope connected to a camera, and the area was measured with ImageJ (v. 2.9.0) after conversion to 8-bit, and thresholding to select only the area of the spheroid. Both day-2 and day-0 area values were converted to µm^2^ in order to calculate the relative area as the ratio between day-2 and day-0. The alignment of the collagen fibers was calculated using the CurveAlign software (v. 5.0) [24]. First, the CT-FIRE module was used to identify the collagen fibers in each z-plane. Then, CurveAlign was used in the CT-Fire Segment mode to remove noise from the image and enhance the fiber edges through the curvelet transform, and then identify the fiber network through a fiber tracking algorithm. Subsequently, fiber orientation with respect to the spheroid edge was measured (boundary analysis). In particular, the mask of the core of the spheroid was provided to the software, which then calculates the relative angle of the fibers with the tangent to the closest point of the boundary. The relative angles were categorized in sectors of 5 degrees, from 2.5 to 87.5, with 2.5 being almost parallel to the tangent to the boundary, and 87.5 being perpendicular to it. Finally, output data from all the z-planes of at least 4 spheroids, in 2 biological replicates, were combined and analyzed with R Studio (v. 2024.04.2–R v. 4.4.1) to create polar plots, showing the distribution of the % of the relative angles for each cell line. For each spheroid, the number of fibers for each degree range was summed through all the z-planes of one spheroid. Then, the % of fibers in each degree range over the total number of fibers was calculated. GraphPad Prism version 9 (Intuitive Software for Science, San Diego, CA, USA) was used for displaying the % of aligned fibers per spheroid, which we defined as the % of fibers with relative angles between 72.5 and 90 degrees.

### 2.9. Statistical Analysis

All the pillar experiments were performed at least in biological triplicate with more than 20 cells analyzed per experimental run. A Wilcoxon test was performed in R Studio to compare cell spreading area and mean force per pillar among the different conditions; linear regression was assessed in R Studio. The statistical significance of the % of nuclear YAP and metastatic separation of mean force/pillar were assessed with GraphPad Prism using one-way ANOVA multiple comparison Tukey’s test. The statistical significances of migration velocity, diffusive fraction, diffusion constant, collagen alignment, and relative area were assessed with GraphPad Prism with ordinary one-way ANOVA multiple comparison test followed by a Šídák’s post hoc test. Statistical significance was set at *p* < 0.05 and is indicated by *, *p* < 0.05; **, *p* < 0.01; ***, *p* < 0.001; ****, *p* < 0.0001, “ns” means not significant.

## 3. Results

**Figure 1:**
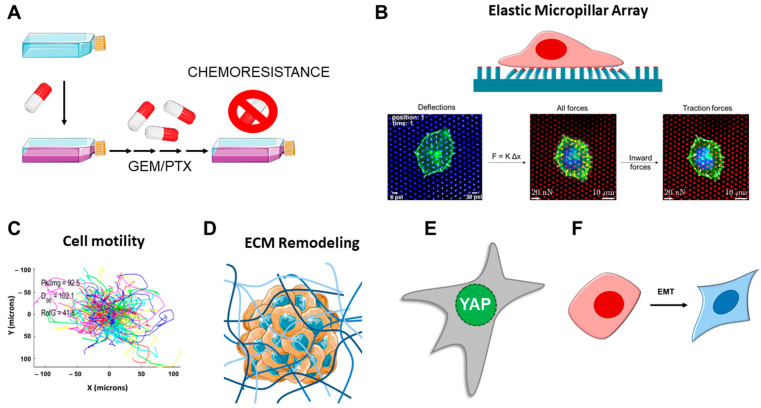
Workflow of the study. (**A**) PDAC cells were exposed to gemcitabine (GEM) or paclitaxel (PTX) to generate chemoresistant clones. (**B**) PDAC cells, and their resistant subclones, seeded on elastic micropillar arrays of varying stiffness were assessed for force generation by measuring the pillar deflections. Traction forces were defined as the inward-pointing forces. (**C**) Single-cell motility was assessed in cells seeded on collagen- and fibronectin-coated substrates. (**D**) The 3D collagen-embedded spheroid invasion and spheroid-induced ECM remodeling were analyzed. (**E**) YAP nuclear translocation assessed by immunofluorescence for cells growing on soft pillars. (**F**) Biomarkers of epithelial-to-mesenchymal transition (EMT) were assessed by RT-qPCR. Part of the figure was adapted from images made by Servier Medical Art by Servier, licensed under a Creative Commons Attribution 4.0 Unported License, at https://smart.servier.com.

In order to elucidate the mechanical properties of PDAC cells, we selected a panel of commercially available cell lines, assessing their force generation using elastic μPAs of varying stiffness. Subsequently, we generated a panel of chemoresistant cells and evaluated whether those have altered mechanical characteristics in terms of force generation, YAP nuclear translocation, EMT, single-cell motility, and 3D invasion capacity in collagen (see the analysis pipeline in Figure 1).

We selected a heterogeneous panel of commercially available cell lines including one non-tumor cell line, HPDE (human pancreatic ductal epithelial); three epithelial-phenotype cell lines BxPC-3, SUIT-2.028, and CAPAN-1 (with BxPC-3 being *KRAS* wild-type); and one mesenchymal-phenotype cell SUIT-2.007. μPA of varying stiffness, coated with fibronectin, were employed to assess cellular force generation. The heights of the pillars were 3.2, 4.1, 6.1 and 6.9 μm, which resulted in a variation in the effective stiffness of the surface of 142, 47, 29 and 11 kPa (Young’s modulus), respectively [20].

### 3.1. The Spreading Area of PDAC Cells Varies Among Cell Lines, and with Substrate Stiffness

**Figure 2:**
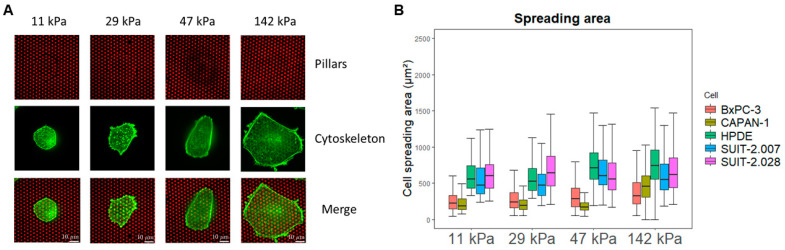
PDAC cell spreading area varies with stiffness. (**A**) Representative confocal microscopy images of BxPC-3 cells (green) growing on fibronectin-coated pillars (red) of different stiffness. Scale bar: 10 µm (**B**) Boxplots of cell spreading area (μm^2^) for BxPC-3, CAPAN-1, HPDE, SUIT-2.028, and SUIT-2.007 growing on fibronectin-coated pillars (25th and 75th percentiles marked, line at median).

We first evaluated whether the cell spreading area was affected by pillar stiffness, and whether different cell lines had similar spreading areas. As has been observed for a variety of cell lines [25,26], we confirmed that PDAC cells also assume a larger spreading area on stiffer plating conditions (Figure 2 and Supplemental Table S2). Additionally, three cell lines, HPDE and SUIT-2.007/028, showed a larger area than CAPAN-1 and BxPC-3 in all the stiffness conditions (Figure 2B). The PDAC cells that had a larger spreading area consequently deflected more pillars.

### 3.2. PDAC Cell Force Generation Is Stiffness-Dependent

**Figure 3:**
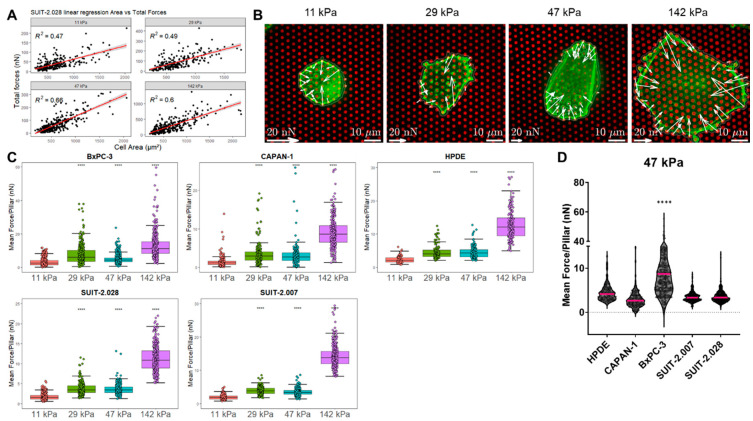
PDAC cell traction forces increase with substrate stiffness, but not with metastatic potential. (**A**) Representative linear regression of the total forces (nN) vs. spreading area (μm^2^) of SUIT-2.028. All the linear regression models of the other cell lines are found in Supplemental Figure S1. In all the measurements, the regression coefficient was R^2^ > 0.45. (**B**) Representative confocal microscopy images of the traction forces of the BxPC-3 cells (green) growing on fibronectin-coated pillars (red). White arrows indicate cellular traction forces on the pillars. (**C**) Boxplots of the PDAC cell traction forces expressed as mean force per pillar (nN) (25th and 75th percentiles marked, line at median). Statistical significance was calculated using the softest condition (11 kPa) as the reference group. (**D**) Mean force per pillar (nN) of the PDAC cell lines of different phenotypes. The results from other stiffness values (11, 29, and 142 kPa) are shown in Supplemental Figure S3 and pillar‘s background forces are shown in Supplemental Figure S2. Each dot of plots in (**A**,**C**,**D**) represents the result from one cell. (**C**,**D**) Statistical significance was set at *p* < 0.05 and is indicated by ****, *p* < 0.0001.

The total force applied by one cell is determined by the sum of all the forces on all the deflected pillars beneath the cell spreading area. In order to compare force generation among the different cell lines and stiffness conditions, the effect of the varying spreading area needed to be excluded. We found that the total force per cell linearly increased with the spreading area of the cell. Quantitatively, this assumption was confirmed by the high linear regression coefficient of R^2^ > 0.45 in all the cases (Figure 3A and Figure S1A). Therefore, we reasoned that the mean force per pillar, which takes into account the number of deflected pillars depending on the spreading area, resulted in a robust (R^2^ < 0.01) and unbiased measure for cellular force generation (Supplemental Figure S1B). In what follows, traction forces are reported as mean force per pillar under the cell.

For all the cell lines studied, force generation increased with pillar stiffness (Figure 3B,C), corroborating findings on other cell lines in which a likewise stiffness-dependent response was observed [25,27]. For instance, in the BxPC-3 cell line, the mean force per pillar increased from 3.2 ± 0.1 nN (mean ± sem) at a substrate stiffness of 11 kPa, to 13.1 ± 0.5 nN at 142 kPa (Figure 3C and Supplemental Table S3).

Next, we assessed whether the cellular phenotype correlates with increased traction forces. It has been described that prostate, breast, and lung metastatic cancer cells show an increased single-cell force generation compared to their respective epithelial-phenotype cells [12]. However, for our panel of PDAC cells, we did not observe the same pattern. When grown on substrates of the same stiffness, the non-tumor cell line HPDE and the epithelial cells CAPAN-1 and SUIT-2.028 showed similar traction forces as compared to the metastatic cells SUIT-2.007 (Figure 3D and Supplemental Table S3). Moreover, the epithelial-like cell line BxPC-3 showed a wide fluctuation in forces, reaching the highest values compared to all the other cell lines investigated.

### 3.3. Chemoresistant PDAC Cells Display an Altered Force Generation

Whether PDAC cells with acquired chemoresistance have an altered mechanical characteristic has so far not been investigated. In particular, to date, no information is available for PDAC paclitaxel-resistant models. The latter is of interest for mechanobiological studies, as the drug paclitaxel affects microtubule polymerization and cytoskeleton rearrangements. We speculated that continuous exposure to paclitaxel could alter the mechanobiology of PDAC cells. Therefore, we investigated in three gemcitabine-resistant (GR) and three paclitaxel-resistant (PR) cells [19] whether force generation was affected by acquired drug resistance and the concurrent genetic changes that come with resistance. The cell line SUIT-2.028 has an epithelial-like phenotype, while PATU-T and SUIT-2.007 were representative of the mesenchymal phenotype. Given that no significant difference was observed between the intermediate stiffness values (29–47 kPa), and that the stiffest value investigated (142 kPa) had less physiological relevance for PDAC, in what follows, we proceeded to compare the soft and stiff substrates of 11 and 47 kPa only. We first confirmed, as for the non-resistant cells, that PDAC chemoresistant cells had variable cell spreading area, and that the mean force per pillar was a faithful measure of traction forces (see Supplemental Table S4 and Supplemental Figure S4).

**Figure 4:**
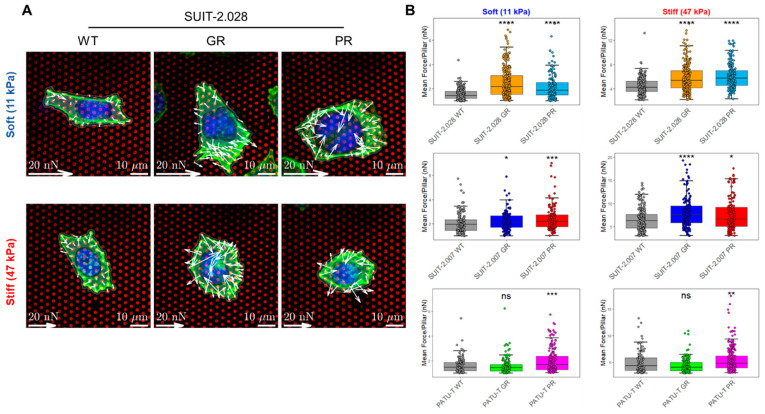
Chemoresistant PDAC cells apply higher traction forces. (**A**) Representative confocal microscopy images of the traction forces of SUIT-2.028 WT, GR, and PR cells growing on fibronectin-coated soft (11 kPa) and stiff (47 kPa) pillars. White arrows indicate traction forces that the cells applied to deflect the pillars. Nucleus is indicated by cyan color (DAPI) and cytoskeleton by green color (AlexaFluor568 Phalloidin). (**B**) Boxplots of PDAC cell traction forces on the soft and stiff pillars on the left and right, respectively, expressed as mean force per pillar (nN) (25th and 75th percentiles marked, and line at median). Statistical significance was calculated using the parental cells (WT) as the reference group. Each dot represents one cell analyzed. Statistical significance was set at *p* < 0.05 and is indicated by *, *p* < 0.05; **, *p* < 0.01; ***, *p* < 0.001; ****, *p* < 0.0001, “ns” means not significant.

Force generation was evaluated on the soft and stiff elastic μPAs of 11 kPa and 47 kPa, respectively. For all cell lines, we observed an increased force generation for paclitaxel-resistant (PR) vs. the parental (WT) cells, independent of stiffness (Figure 4 and Supplemental Table S5). For the gemcitabine-resistant (GR) cells, both the SUIT-2.028 and SUIT-2.007 cells applied higher traction forces as compared to the parental clones. On the soft substrate (11 kPa), the mean force per pillar was 1.5 ± 0.1 nN (SUIT-2.028 WT, mean ± sem) vs. 2.5 ± 0.1 nN (SUIT-2.028 GR) and 2.0 ± 0.1 nN (SUIT-2.007 WT) vs. 2.2 ± 0.1 nN (SUIT-2.007 GR). On the stiff substrate (47 kPa), the mean force per pillar was 4.5 ± 0.1 nN (SUIT-2.028 WT) vs. 5.9 ± 0.1 nN (SUIT-2.028 GR) and 6.3 ± 0.2 nN (SUIT-2.007 WT) vs. 8.1 ± 0.3 nN (SUIT-2.007 GR). However, no significant difference was observed for PATU-T GR and WT in both stiffness conditions (Supplemental Table S5). Together, these results indicate that PR cells apply higher traction forces on both the soft and stiff substrates, while in GR-cells, an increase in traction force was observed in SUIT-2.028 and SUIT-2.007, but not in PATU-T.

### 3.4. Chemoresistant PDAC Cells Demonstrate Distinct Migratory Behavior Compared to Their Parental Cells

Migration and force application are tightly intertwined, as they both rely on the activity of the cytoskeleton [28]. Given the change in force application observed in resistant cells, we wondered whether those would be likewise reflected in the migration and invasive potential of the chemoresistant phenotypes in a single-cell motility assay. Cell nuclei were stained with Hoechst 33342 and exposed to 5 µM Verapamil for 1 h. Verapamil was used to inhibit the efflux of Hoechst, as we earlier found that PR cells overexpress the membrane transporter ABCB1 [19], and thus would quickly lose their Hoechst staining. Before cell plating, substrates were coated by either collagen or fibronectin, as those ECM proteins represent the most abundant proteins in the PDAC microenvironment [29,30]. The stained nuclei were subsequently imaged with confocal fluorescent microscopy at intervals of 12 min for 16 h. Multiple parameters such as single-cell velocity, diffusion constant, diffusive fraction, and directionality were extracted from tracking individual cells over time (for details, see the Supplementary Material).

**Figure 5:**
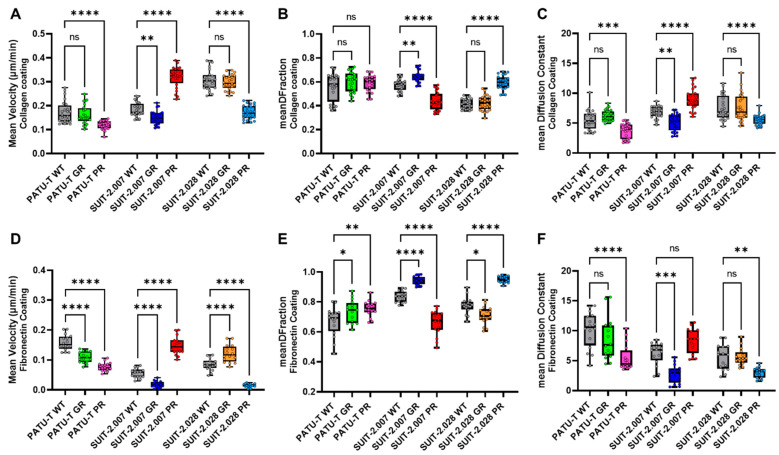
PDAC resistant single-cell migration is different from parental cells. PDAC single-cell mean velocity (μm/min), growing on (**A**) collagen-coated or (**D**) fibronectin-coated wells. The directionality of PDAC cell migration trajectories growing on collagen-coated wells, expressed as (**B**) diffusive fraction = DF and (**C**) diffusion constant. The directionality of PDAC cell migration trajectories growing on fibronectin-coated wells, expressed as (**E**) DF fraction and (**F**) diffusion constant. All the conditions are represented as boxplots with the smallest and largest values marked, and line at median. The statistical significance was calculated using the parental cells (WT) as the reference group. Each dot represents the population mean for one section of the well. (**A**–**F**) Statistical significance was set at *p* < 0.05 and is indicated by *, *p* < 0.05; **, *p* < 0.01; ***, *p* < 0.001; ****, *p* < 0.0001, “ns” means not significant.

The velocity of chemoresistant PDAC cells was, in general, different from their parental clones (Figure 5A,D). The GR-resistant cells were characterized by a slight reduction in their velocity on both substrates. Paclitaxel resistance resulted in a more pronounced, yet also clear cell-specific response. For PATU-T and SUIT-2.028 PR cells, the velocity almost halved compared to the parental cells (WT) on both substrates. Conversely, for SUIT-2.007 PR, the velocity almost doubled, consistent for both substrates. It appeared that velocity, which characterizes the active and directed part of cell motility, is clearly, yet differentially, altered in chemoresistant cells.

A measure of the general activity of cells is their diffusional motility characterized by a diffusion constant. In our experiments, the diffusion constant followed the pattern of the velocity (Figure 5C,F). It should be noted that treatment by 5 µM Verapamil to block the ABCB1 transporter did not affect the migration patterns (Supplemental Figure S5A), while the positive control, bosutinib, which is known to efficiently inhibit migration (Supplemental Figure S5B), resulted in velocity values close to zero.

Given that cell motility was characterized by two modes, active directed motion and diffusion, we further analyzed which of the two modes dominated. For that, we defined a diffusive fraction (*DFraction*), which quantifies the fraction of diffusion on the total mean-squared displacement at a time lag of 240 min. *DFraction* approaches unity for purely diffusive motion and reaches zero for purely directed motion. For all cells and conditions, mobility was diffusion-dominated with *DFraction* > 0.5 (Figure 5B–E). For most conditions, the diffusive fraction only gradually changed, with a pronounced difference for two cell lines resistant to paclitaxel. In detail, where SUIT-2.028 PR cells lost all the active directional part of their motility (*DFraction* = 0.59 ± 0.01), SUIT-2.007 PR cells’ motility increased the active, directional part of their motility (*DFraction* = 0.44 ± 0.02) (Figure 5B,E).

### 3.5. Migration and Force Application of PDAC Cells in a 3D Extracellular Matrix

**Figure 6:**
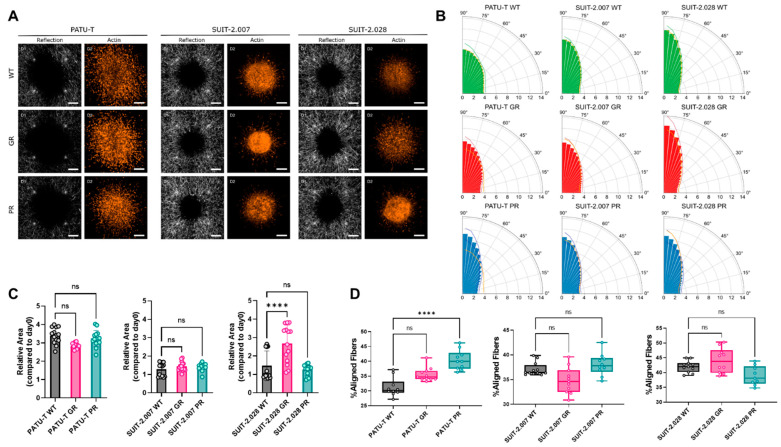
PDAC chemoresistant cell ability for 3D ECM remodeling and invasion. (**A**) Representative collagen fibers alignment (left columns), obtained by reflection microscopy, and actin (right columns) of PDAC chemoresistant spheroids. Scale bar: 200 µm (**B**) Polar plots representing the percentage of the frequency of the distribution of collagen fibers angles from 0° to 90°. Each bar represents the % of fibers in a sector of 5 degrees, expressed as the mean of 2 biological replicates, with at least 4 technical replicates. Lines of the darker shade of the bars represent the upper and lower bounds of SD of the % of collagen fibers in each sector. Orange lines represent the mean % of the frequency of the respective WT for each cell line to facilitate the comparison. (**C**) Relative area covered by spheroids after 2 days. Dots represent the value of individual spheroids. Data are expressed as mean ± SD. (**D**) Percentage of aligned fibers, defined as fibers comprised between the angles of 72.5 and 90, which means that those fibers are perpendicular to the closest point of the spheroid. Dots represent the value of individual spheroids. (**C**,**D**) Statistical significance was set at *p* < 0.05 and is indicated by ****, *p* < 0.0001. “ns” means not significant.

Physiologically, tumor cells move in the three-dimensional tumor microenvironment (TME). In the TME, cells come into contact with extracellular matrix proteins (collagen, hyaluronic acid, laminin, fibronectin, and others), and with other types of cells (fibroblasts, immune cells, and others). Here, we mimicked this complex in vivo situation by spheroids of each cell line which were micro-injected in type-1 collagen hydrogels to study the behavior of PDAC cells in a three-dimensional (3D) context resembling the TME. 24 h after injection, collagen fibers alignment was measured as the resemblance of cellular force generation with confocal reflection microscopy. After 48 h, 3D cell migration was measured as the ratio between the area of the spheroid in z-projection between day 2 and day 0 (Figure 6A). Resistant cell motility in 3D did not significantly differ from their parental clones, with the exception of SUIT-2.028 GR. Specifically, for the SUIT-2.028 GR cells, the relative spheroid area was significantly larger compared to SUIT-2.028 WT (2.5 ± 0.9 vs. 1.5 ± 0.9) (Figure 6C and Supplemental Table S6).

The differential force application that we observed in 2D on the µPAs predicted a differential ability of cells in aligning/remodeling collagen fibers around the spheroids. We, therefore, measured the angle of the fibers with the closest point to the core of the spheroid. Before micro-injection, the collagen fibers were randomly oriented (Supplemental Figure S6). We considered the fibers as aligned for angles between 72.5 and 90 degrees with respect to the tangent of the spheroid outline. Only PATU-T PR caused a significant increase in collagen alignment despite applying more forces than the WT on the pillar for all three cell lines (Figure 6B,D). We also observed a trend towards increased collagen alignment in SUIT-2.028 GR and PATU-T GR (Figure 6D). However, there was a clear difference in the fraction of aligned fibers between the three different parental cell lines, with SUIT-2.028 WT aligning more fibers than SUIT-2.007 and PATU-T (Figure 6B,D).

### 3.6. YAP Nuclear Translocation and EMT Are Not Related to Increased Traction Forces

**Figure 7:**
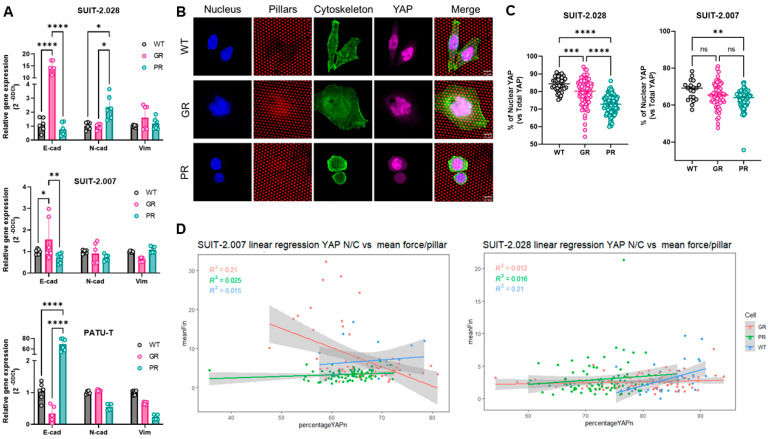
PDAC chemoresistant cell differential mechanobiology does not rely on YAP nuclear translocation nor on EMT switch. (**A**) Relative gene expression of *E-cadherin*, *N-cadherin*, and *Vimentin* as assessed by RT-qPCR. Data are expressed as the mean ± SD of three independent experiments. (**B**) Representative confocal microscopy images of SUIT-2.028 cells growing on soft (11 kPa) pillars and stained with YAP. Scale bar: 10 µm (**C**) YAP nuclear translocation, expressed as % of nuclear YAP over total YAP in SUIT-2.028 and SUIT-2.007. (**D**) Linear regression model between the mean force per pillar vs. % of nuclear YAP in SUIT-2.007 (left panel) and SUIT-2.028 (right panel). Each dot in (**C**,**D**) represents one cell. *, *p* < 0.05; **, *p* < 0.01; ***, *p* < 0.001; ****, *p* < 0.0001, “ns” means not significant.

To characterize mechano-response in chemoresistant cells, we checked whether differences in traction forces and migration patterns were paralleled by epithelial-to-mesenchymal transition (EMT), and the occurrence of nuclear yes-associated protein (YAP) translocation. EMT is the classical de-differentiation process epithelial cancer cells undergo, resulting in a more aggressive and invasive phenotype. EMT is characterized by a change in the expression of specific genetic markers among which is a reduced expression of *E-cadherin* (*E-cad*), and an increase in *N-cadherin* (*N-cad*) and *Vimentin* (*Vim*) expression. Interestingly, when comparing the expression of the abovementioned markers in GR and PR clones vs. parental cells (WT), a cell line-dependent effect was observed. PATU-T PR displayed an epithelial switch, with the reduced expression of both *N-cad* and *Vim,* and a 60-fold increase in *E-cad* expression (Figure 7A). Similarly, SUIT-2.007 GR showed a decreased expression of *Vim*, and a trend towards increased *E-cad* (Figure 7A). However, SUIT-2.028 GR showed a 15-fold increase in *E-cad* expression, while SUIT-2.028 PR an increase in *N-cad* (Figure 7A). Collectively, no consistent pattern was observed for the EMT switch among different cell lines, which could explain the increased force generation measured for chemoresistant cells.

Next, we investigated YAP nuclear translocation. This process is triggered by the mechanical stimuli of cells, and marks an active mechanobiology status of the cell [31,32]. Previous studies showed that YAP nuclear translocation is triggered on stiff substrates in mesenchymal stem cells or breast cancer cells [33,34]. Therefore, we analyzed the level of YAP in the nucleus on the softest pillar arrays (11 kPa) to exclude that all YAP was translocated due to stiffness response. Surprisingly, despite both SUIT-2.028/007 GR and PR cells applying more force than their parental counterparts (WT), we did not find an increase in YAP translocation in the nucleus (Figure 7B,C). Conversely, both GR and PR cells showed less YAP in the nucleus compared to WT cells (Figure 7C). To exclude that the increased traction forces are directly related to YAP nuclear translocation, we analyzed the correlation between those two quantities in a linear regression model. Interestingly, we found no correlation (R^2^ ≤ 0.2) between cellular traction forces and YAP translocation (Figure 7D). Together, our results indicate that the increased traction forces generated by chemoresistant cells do neither rely on EMT nor on YAP signaling.

## 4. Discussion

This is the first study reporting an extensive characterization of the mechanobiological features of PDAC chemoresistant cells, indicating that paclitaxel- and gemcitabine-resistant cells apply higher forces, and their motility in 2D and 3D differs from their parental clones.

PDAC acquired chemoresistance is the major cause of poor patient prognosis. Upon drug treatment, cancer cells adapt to evade drug-mediated cell death by modifying signaling pathways, the gene expression of drug transporters, and other mechanisms [35,36]. However, a better understanding of these biological mechanisms does not fully cover the knowledge of the altered cellular processes of PDAC chemoresistance. More precisely, mechanobiology and physical forces play an important role in mediating PDAC chemoresistance. In particular, cells being periodically exposed to cytoskeleton disruptor molecules (e.g., paclitaxel, a microtubule disassembly inhibitor) could acquire differential mechano-transduction patterns. As such, in a previous study by our group, we focused on the most widely employed drug for PDAC, i.e., gemcitabine. In this study, we reported that PDAC cells acquire resistance to gemcitabine when cultured on stiff substrates, and that cells with acquired chemoresistance have an overexpression of the ECM-binding integrin-α2 (ITGA2) [10].

**Figure 8:**
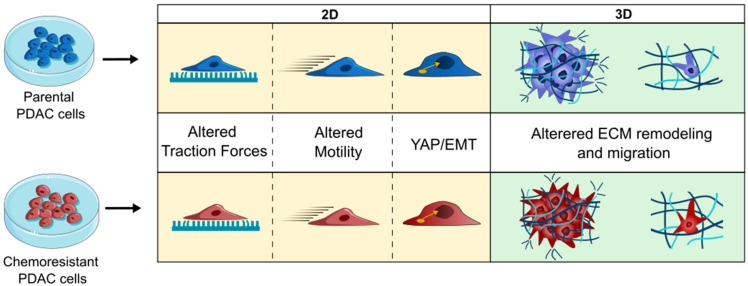
PDAC resistance leads to changes in the mechanobiological signatures of cells, the detailed characteristics of which yet depend on cell type and ECM dimensionality. Part of the figure was adapted from images made by Servier Medical Art by Servier, licensed under a Creative Commons Attribution 4.0 Unported License, at https://smart.servier.com.

The current study further explored the altered mechanical properties, which we summarize here as mechanobiology, of PDAC cells with acquired resistance to either gemcitabine or paclitaxel, two commonly used drugs in PDAC treatment. Remarkably, we observed that PDAC cells apply more traction forces on stiffer substrates and, upon acquired chemoresistance to either of the drugs, PR or GR showed consistently higher force generation compared to their parental cells. Moreover, chemoresistant cells showed differences in migration, as well as in 3D collagen remodeling and in 3D invasion. However, these altered mechanical and motility features were not reflected by previously reported biological processes, such as an increased YAP nuclear translocation or an EMT switch. A graphical representation of our findings is displayed in Figure 8. 

In the current study, we used elastic micropillar arrays to measure traction forces generated by a panel of PDAC cell lines. The methodology allowed us to measure cellular contractile forces with a precision below 1 nN [20,37]. When grown on pillars of higher stiffness, the PDAC cells displayed a larger spreading area, a finding that is in line with previous studies on fibroblast and endothelial cells using the same experimental settings [21,38]. Additionally, previous studies reported that for fibroblast and other cell types, cell traction forces increase with substrate stiffness [25,26,27,39]. Here, we validated both findings in five PDAC cell lines. Of note, our results were independent of whether cells had an epithelial or mesenchymal phenotype. Furthermore, here we report that PDAC cells with acquired chemoresistance to gemcitabine and paclitaxel adopt an increased contractile behavior as compared to their parental clones. Previous studies mainly focused on investigating PDAC chemoresistance triggered by culturing drug-sensitive cells on substrates with varying stiffness [9,10,40,41]. For instance, Shah and collaborators characterized some mechanical features of PDAC cells with acquired gemcitabine resistance [15], showing a switch to mesenchymal phenotype and an increased migratory/invasive potential. Our results did not validate those findings. This controversy may be explained by variations in experimental conditions. To closely emulate native settings, we indeed cultured cells on ECM-coated substrates and controlled near-native stiffness conditions.

EMT and cellular force generation are believed to be closely correlated. For example, metastatic/mesenchymal cancer cells of different tumor types (prostate, breast, and lung) showed higher contractile forces [7,12] compared to non-metastatic phenotypes. The correlation between EMT and traction forces in PDAC was first investigated by Nguyen and collaborators, who reported that in PDAC, the metastatic potential does not correlate with increased traction forces. The PDAC mesenchymal cell line Hs766 applied less traction forces compared to the epithelial/quasi-mesenchymal PANC-1. Those findings were, in part, corroborated by our results. The epithelial cell line BxPC-3 applied higher forces compared to the mesenchymal SUIT-2.007 cells. However, the non-tumor cells HPDE as well as the epithelial cells (CAPAN-1 and SUIT-2.028) showed no significant differences in traction forces as compared to the mesenchymal cells. This could be, at least in part, explained by the hypothesis proposed by Nguyen and collaborators, suggesting that rather than the phenotype, it is the activity of myosin II signaling that orchestrates cellular stiffness and invasion [5].

Prompted by these interesting and contrasting results, we, therefore, investigated whether the cells with acquired chemoresistance had an altered EMT status. We investigated the mesenchymal cell lines PATU-T and SUIT-2.007, as well as the epithelial SUIT-2.028 cells, which, despite their origin from a metastatic site, are characterized by a more epithelial phenotype. Regarding traction forces, SUIT-2.007 exhibited the highest forces among all the cell types. Yet, PATU-T had lower forces compared to SUIT-2.028. We subsequently investigated whether changes in the expression of EMT genes could explain the observed differences in traction forces between the WT and resistant cells. Surprisingly, we did not observe a distinct pattern across the cell lines. For some of the cells that showed higher contractile forces, there was a tendency towards an epithelial phenotype switch. Contrarily, a previous study reported a shift towards a mesenchymal status for the gemcitabine-resistant PDAC cells L3.6pl GR, being characterized by a decreased expression of *E-cadherin* and an increase in *Vimentin* [15]. Overall, our findings contradict the preliminary notion that contractile forces and invasive potential correlate with a more mesenchymal phenotype [10,12,14]. This might be explained by the different cellular models employed. In our study, we validated results using multiple cell lines with different phenotypes (epithelial and mesenchymal), which provides more robust evidence for our results.

Another robust indicator of activated mechano-transduction in cells is the translocation of the transcription factor YAP to the nucleus [31]. Previous studies reported that cells growing on stiffer substrates and with an increased invasive potential showed an elevated YAP nuclear localization [33,34]. Intriguingly, in our study, chemoresistant cells that applied higher forces showed less YAP nuclear translocation compared to the parental clones, with no observable correlation with increased contractile forces. This result could be caused by the use of different experimental models. It should be noted that the measurement of YAP localization was conducted on only one stiffness condition, and the comparison is between the diverse cell types. YAP machinery is complex; hence, different stiffnesses, geometries, and cell lines chosen could lead to different results [34]. Further studies could elucidate the impact of substrate stiffness on YAP localization in PDAC chemoresistant cells.

Cell motility is a crucial parameter to characterize the ability of cells to invade tissue during metastatic dissemination. Motility and migration are largely influenced by the surrounding ECM composition and the rheological properties of tissue [11]. We previously reported that PDAC cells showed increased migration and invasion features when growing as a monolayer on the collagen-coated substrates of controlled stiffness [9]. In the present study, we further explored the role of ECM coating by analyzing single-cell motility on either collagen or fibronectin-coated substrates, the two most abundant ECM proteins found in PDAC. Interestingly, we observed that all the chemoresistant cells and their parental clones had the same mobility pattern independently of substrate coating. This finding suggests that PDAC chemoresistant cells underwent some intrinsic (mechano)biological modification, allowing them to migrate differently, rather than adapting to the different ECM substrate. However, when embedded in a 3D matrix, a more controversial behavior was observed: cells appeared confined within the collagen matrix and showed a slower invasion rate. We did not find a consistent pattern for the PDAC chemoresistant cell lines. In 2D, the PATU-T cell line exhibited the slowest migration, whereas in 3D, the covered area was larger than that of SUIT-2.007 and SUIT-2.028, suggesting that PATU-T cells moved faster through the matrix. One plausible explanation could be attributed to the experimental settings, given the different timescales investigated to measure migration in 2D and 3D environments. The results from spheroids (3D migration) suggest that changes in the covered area were related to the different parental cell line rather than the drug used to establish resistance.

Regarding the ability to align collagen, gemcitabine resistance led to an increased fiber alignment in the SUIT-2.028 GR cells, but a decrease in the SUIT-2.007 GR compared to the WT. Similarly, the establishment of paclitaxel resistance resulted in PATU-T PR aligning more fibers, while SUIT-2.028 PR cells exhibited fewer aligned fibers compared to the respective parental cells. It is noteworthy that the GR and PR clones of the same cell line, e.g., SUIT-2.028, had opposite collagen aligning abilities when compared to their parental clones. Furthermore, we observed that the ability to align collagen in 3D and the force application on μPAs in 2D do not follow the same trend. Cells applying more forces in 2D are not always able to invade more in 3D, nor are they able to align collagen fibers. This could be in part due to the significant differences in the scaffold stiffness in the two assays. The 2D data have been collected on supports of at least 11 kPa, while collagen hydrogels are typically below 100 Pa [42,43,44]. This difference in stiffness could influence the magnitude of force application, as we observed that higher stiffness can trigger an increase in force application. Moreover, it is important to note that the 3D matrices employed are simplistic representations of the PDAC tumor microenvironment, which has more ECM proteins than only collagen, thus possibly affecting the differential behavior observed. Not only the stiffness, but also the different types of models, such as a 2D flat surface versus a 3D matrix with pores, can trigger different types of migration [45], which might not retain the same characteristics. Therefore, the exact mechanism of the controversial behavior in 2D and 3D needs to be elucidated in further studies. NF-E2-related factor 2 (Nrf2) might offer an interesting direction for future studies, as its connection with chemoresistance was previously shown in PDAC and other cancers [46,47]. It is a transcription factor normally degraded and activated in case of oxidative and electrophilic stress [48]. In *KRAS*-mutated cancers, NRF2 can be increased [46], and a study in NSCLC showed how NRF2 can also regulate cell motility independently from EMT [48]. Interestingly, it affects RhoA/ROCK1 signaling, which is related to force application and stress-fiber formation.

It is important to note that despite the shared mechanism of resistance development, i.e., the overexpression of ABCB1 transporter [19], force application and the migration of PR PDAC cells are not affected in the same way by prolonged exposure to the drug.

## 5. Conclusions

In conclusion, our study characterized multiple mechanical features of PDAC chemoresistant cells and highlighted the importance of including multiple cell models when studying complex physical and biological behaviors. We here reported that to evaluate the effect of drug perturbation on the physical parameters of tumors, heterogeneity plays a crucial role. Therefore, models that more closely resemble the physiological and physical characteristics of the tumor microenvironment, like tumoroids in a close-to-native TME environment, need to be adopted for a deeper understanding of PDAC mechanobiology.

## Data Availability

All the experimental datasets and documents generated in this study are available upon reasonable request to the corresponding author.

## Supplementary Materials

The following supporting information can be downloaded at https://www.mdpi.com/article/10.3390/cancers16223863/s1, Figure S1: Linear regression model of (A) total forces (nN) vs. spreading area (μm^2^), and (B) mean force per pillar (nN) vs. spreading area (μm^2^) of PDAC cells; Figure S2: Pillar ‘background forces’; Figure S3: Traction force of PDAC cells. Mean force per pillar (nN) of different PDAC cell lines growing on pillars with varying stiffness. Statistical significance was set at *p* < 0.05 and is indicated by **, *p* < 0.01; ****, *p* < 0.0001.; Figure S4: Linear regression model of the mean force per pillar (nN) vs. spreading area (μm^2^) of PDAC chemoresistant cells grown on (A) soft (11 kPa), and (B) stiff (47 kPa) pillars; Figure S5: PDAC cell migration is effectively inhibited by the motility-inhibitor bosutinib but not affected by the ABCB1-blocker verapamil; Statistical significance was set at *p* < 0.05 and is indicated by *, *p* < 0.05; ***, *p* < 0.001; ****, *p* < 0.0001, “ns” means not significant. Figure S6: Representative confocal reflection images of single z-planes of empty collagen gels, showing the random orientation of collagen fibers; Table S1: List RT-qPCR primers’ sequence; Table S2: Spreading area of PDAC cells; Table S3: Traction forces of PDAC cells; Table S4: Spreading area of PDAC chemoresistant cells; Table S5: Traction forces of PDAC chemoresistant cells; Table S6: Mean relative area of PDAC spheroids migrating in collagen matrix. References [22,49,50] are cited in the Supplementary Materials.
