# Supplementary material for: Altered Mechanobiology of PDAC Cells with Acquired Chemoresistance to Gemcitabine and Paclitaxel
Source: Cancers (Basel). 2024 Nov 18;16(22):3863. doi: 10.3390/cancers16223863 (PMC11593083; doi:10.3390/cancers16223863)
Supplement: Supplementary file 1 [file cancers-16-03863-s001.zip › cancers-3271473-supplementary.pdf]

Supplemental Materials to

Altered Mechanobiology of PDAC Cells with Acquired Chemoresistance to Gemcitabine and Paclitaxel

Alessandro Gregori [1,2,3,†], Cecilia Bergonzini [4,†], Mjriam Capula [5], Rick Rodrigues de Mercado [3], Erik H. J. Danen [4], Elisa Giovannetti [1,2,5] and Thomas Schmidt [3,*]

1 Cancer Biology and Immunology, Cancer Center Amsterdam, 1081 HV Amsterdam, The Netherlands

2 Department of Medical Oncology, Amsterdam UMC, Location Vrije Universiteit Amsterdam, 1081 HV Amsterdam, The Netherlands

3 Physics of Life Processes, Huygens-Kamerlingh Onnes Laboratory, Leiden University, 2311 EZ Leiden, The Netherlands

4 Leiden Academic Center for Drug Research, Leiden University, 2311 EZ Leiden, The Netherlands

5 Fondazione Pisana per La Scienza, 56017 San Giuliano Terme, Italy

* correspondence: schmidt@physics.leidenuniv.nl

† These authors contributed equally to this work and share first authorship.

Single-cell motility analysis

The motility of individual PDAC cells was analyzed using a MatLab (MatLab R2018a; MathWorks, Natick, MA, USA) script. For each timepoint images were first thresholded in the respective nuclear marker channel. The center-of-mass positions of all thresholded objects that had the predicted area of a nucleus (10 μm^2< nuclear area < 400 μm^2) were determined. From the center-of-mass position data, 2D cell trajectories were constructed using an assignment algorithm described earlier [1]. The mobility of each cell which was observed for at least for 240 min, was further analyzed in terms of the change in the mean-squared displacement (MSD) with lag-time (t_{lag}) between two time-points.

We considered two types of movement: one involving diffusion, which is characterized by a diffusion constant D, and a second describing directed active motion characterized by a velocity v [1,2]. In this situation the MSD changes with lag-time were calculated as:

$$MSDtlag = 4Dtlag + v2tlag2 \qquad \text{(S1)}$$

In order to characterize the overall motility, we further defined the diffusive fraction f_D, as the ratio of the diffusive part of the $MSD_D(t_{lag})$ = 4 D t_{lag}, to the total MSD, at a fixed lag-time, t_D = 240 min. The diffusive fraction is given by:

$$fD = 11 + v24D \cdot tD \qquad \text{(S2)}$$

Supplemental Table S1. List RT-qPCR primers' sequence.

Gene	Forward/Reverse Sequence	Sequence
**E-cadherin**	Fw	CAATGCCGCCATCGCTTAC
	Rv	ATGACTCCTGTGTTCCTGTTAATG
**N-cadherin**	Fw	GACAATGCCCCTCAAGTGTT
	Rv	CCATTAAGCCGAGTGATGGT
**Vimentin**	Fw	GAGAACTTTGCCGTTGAAGC
	Rv	GCTTCCTGTAGGTGGCAATC

Supplemental Table S2. Spreading area of PDAC cells.

PDAC cells	Stiffness (kPa)	Spreading area (μm^2 mean ± S.E.M.)
**HPDE**	11	598 ± 24
	29	582 ± 22
	47	779 ± 28
	142	788 ± 26
**BxPC-3**	11	274 ± 10
	29	295 ± 10
	47	339 ± 11
	142	402 ± 15
**CAPAN-1**	11	221 ± 7
	29	226 ± 8
	47	195 ± 5
	142	476 ± 16
**SUIT-2.028**	11	664 ± 20
	29	704 ± 20
	47	629 ± 18
	142	694 ± 21
**SUIT-2.007**	11	541 ± 19
	29	518 ± 16
	47	694 ± 23
	142	629 ± 18

Supplemental Table S3. Traction forces of PDAC cells.

PDAC cells	Phenotype	Stiffness (kPa)	Number of cells	Traction Force (nN mean ± S.E.M.)
**HPDE**	Non-tumor	11	76	2,2 ± 0,1
		29	134	4,6 ± 0,2
		47	144	4,5 ± 0,1
		142	163	13,0 ± 0,4
**BxPC-3**	Epithelial	11	357	3,2 ± 0,1
		29	344	7,8 ± 0,3
		47	436	5,8 ± 0,2
		142	307	13,1 ± 0,5
**CAPAN-1**	Epithelial	11	27	1,4 ± 0,1
		29	282	3,7 ± 0,2
		47	288	3,2 ± 0,2
		142	253	9,0 ± 0,3
**SUIT-2.028**	Epithelial	11	248	1,7 ± 0,1
		29	234	3,8 ± 0,1
		47	293	3,6 ± 0,1
		142	293	11,4 ± 0,2
**SUIT-2.007**	Mesenchymal	11	149	2,0 ± 0,1
		29	209	3,9 ± 0,1
		47	228	3,5 ± 0,1
		142	320	14,4 ± 0,2

Supplemental Table S4. Spreading area of PDAC chemoresistant cells.

PDAC cells	Chemoresistance status	Stiffness (kPa)	Spreading area (μm² mean ± S.E.M.)
**SUIT-2.028**	WT	11	428 ± 9
	WT	47	480 ± 12
	GR	11	562 ± 18
	GR	47	555 ± 18
	PR	11	372 ± 18
	PR	47	405 ± 16
**SUIT-2.007**	WT	11	522 ± 19
	WT	47	500 ± 15
	GR	11	418 ± 22
	GR	47	371 ± 14
	PR	11	485 ± 24
	PR	47	482 ± 18
**PATU-T**	WT	11	520 ± 18
	WT	47	518 ± 25
	GR	11	621 ± 21
	GR	47	641 ± 28
	PR	11	556 ± 20
	PR	47	498 ± 14

Supplemental Table S5. Traction forces of PDAC chemoresistant cells.

PDAC cells	Chemoresistance status	Stiffness (kPa)	Number of cells	Traction Force (nN mean ± S.E.M.)
**SUIT-2.028**	WT	11	280	1,5 ± 0,1
	GR	11	289	2,5 ± 0,1
	PR	11	152	2,1 ± 0,1
	WT	47	248	4,5 ± 0,1
	GR	47	284	5,9 ± 0,1
	PR	47	222	5,9 ± 0,2
**SUIT-2.007**	WT	11	158	2,0 ± 0,1
	GR	11	147	2,2 ± 0,1
	PR	11	129	2,4 ± 0,1
	WT	47	218	6,3 ± 0,2
	GR	47	158	8,1 ± 0,3
	PR	47	153	7,7 ± 0,3
**PATU-T**	WT	11	167	1,5 ± 0,1
	GR	11	243	1,3 ± 0,1
	PR	11	212	1,9 ± 0,1
	WT	47	165	4,3 ± 0,2
	GR	47	221	3,9 ± 0,1
	PR	47	253	5,3 ± 0,2

Supplemental Table S6. Mean relative area of PDAC spheroids migrating in collagen matrix.

PDAC cells	Chemoresistance Status	Relative Area (Mean ± S.E.M.)
**PATU-T**	WT	3,4 ± 0,3
	GR	2,8 ± 0,1
	PR	3,2 ± 0,3
**SUIT-2.007**	WT	1,3 ± 0,3
	GR	1,4 ± 0,2
	PR	1,4 ± 0,2
**SUIT-2.028**	WT	1,5 ± 0,6
	GR	2,7 ± 0,7
	PR	1,3 ± 0,2

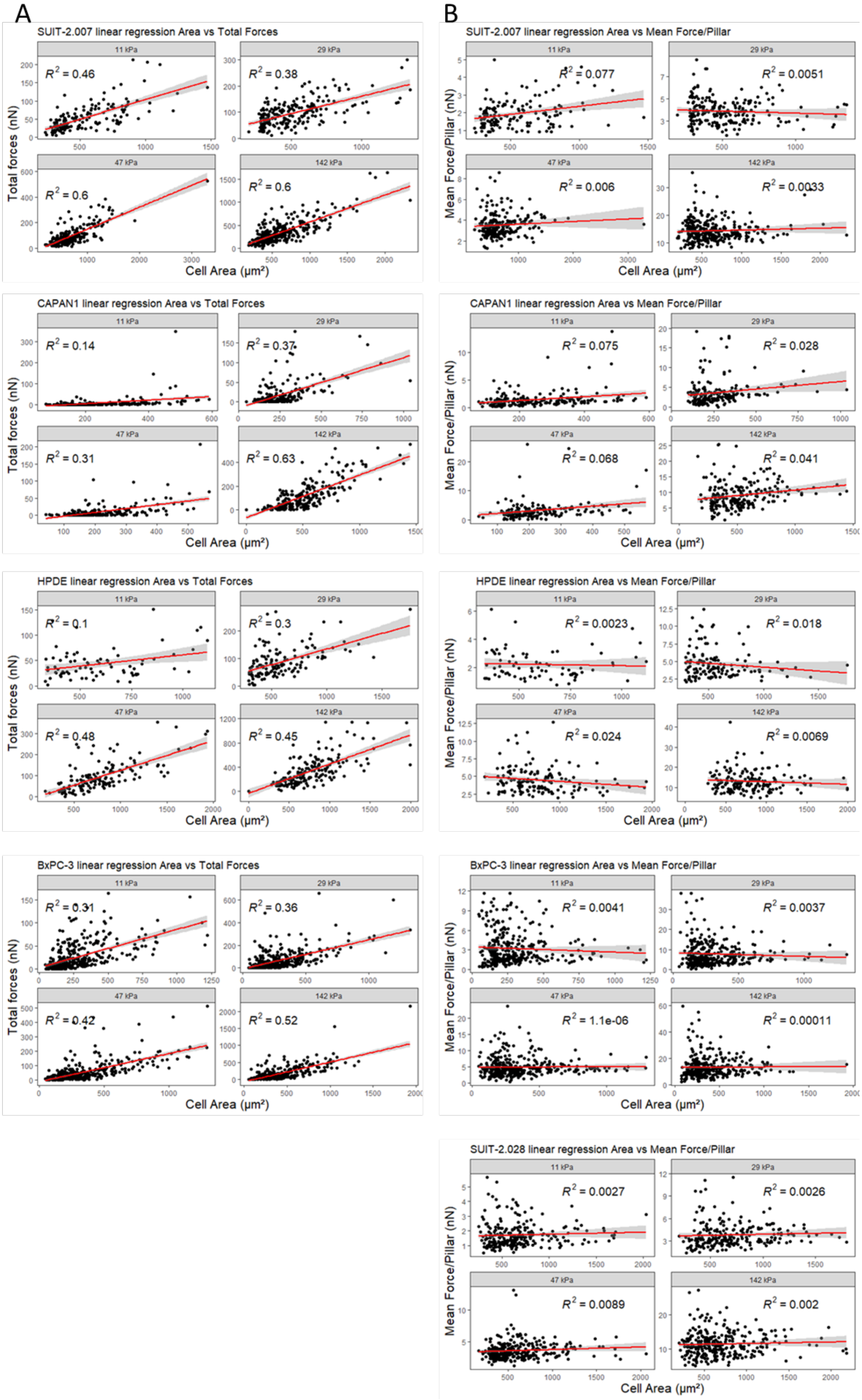

Supplemental Figure S1. Linear regression model of (**A**) total forces (nN) *vs* spreading area (μm^2), and (**B**) mean force per pillar (nN) *vs* spreading area (μm^2) of PDAC cells.

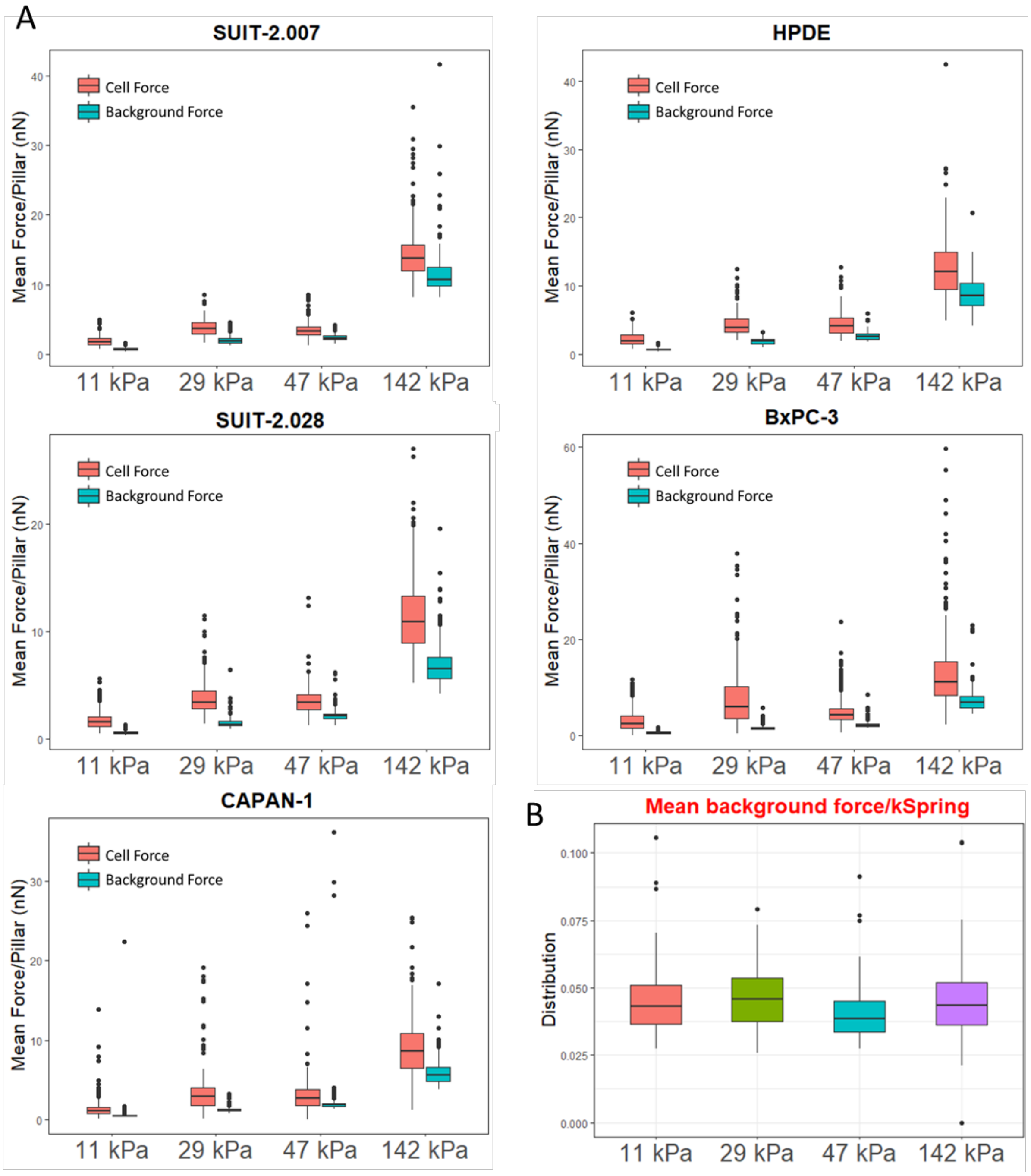

Supplemental Figure S2. Pillar 'background forces'. (**A**) Mean force per pillar (nN) calculated on pillars deflected either under the cell area (orange = cellular force) or outside the cell area (cerulean = 'background force'). The 'background force' is given by the accuracy, at which the center-of-mass of each pillar is determined. It's value is given by the ratio of the pillar diameter (2 µm), and the square-root of the integrated signal for each pillar [3]. In our experiment the integrated signal was ~2000 cnts, which results in an accuracy of pillar detection of ~50 nm. Multiplication with the respective spring constant results in an apparent background-force. Since we report on force magnitude only, the background-force does not vanish but is finite. In all cases, the cellular forces clearly exceed the background. (**B**) When background-forces are divided by the respective spring constants, the resulting displacements had indeed identical distributions of mean of 0.04 ± 0.01 um (mean ± sd), as predicted from the theory [3].

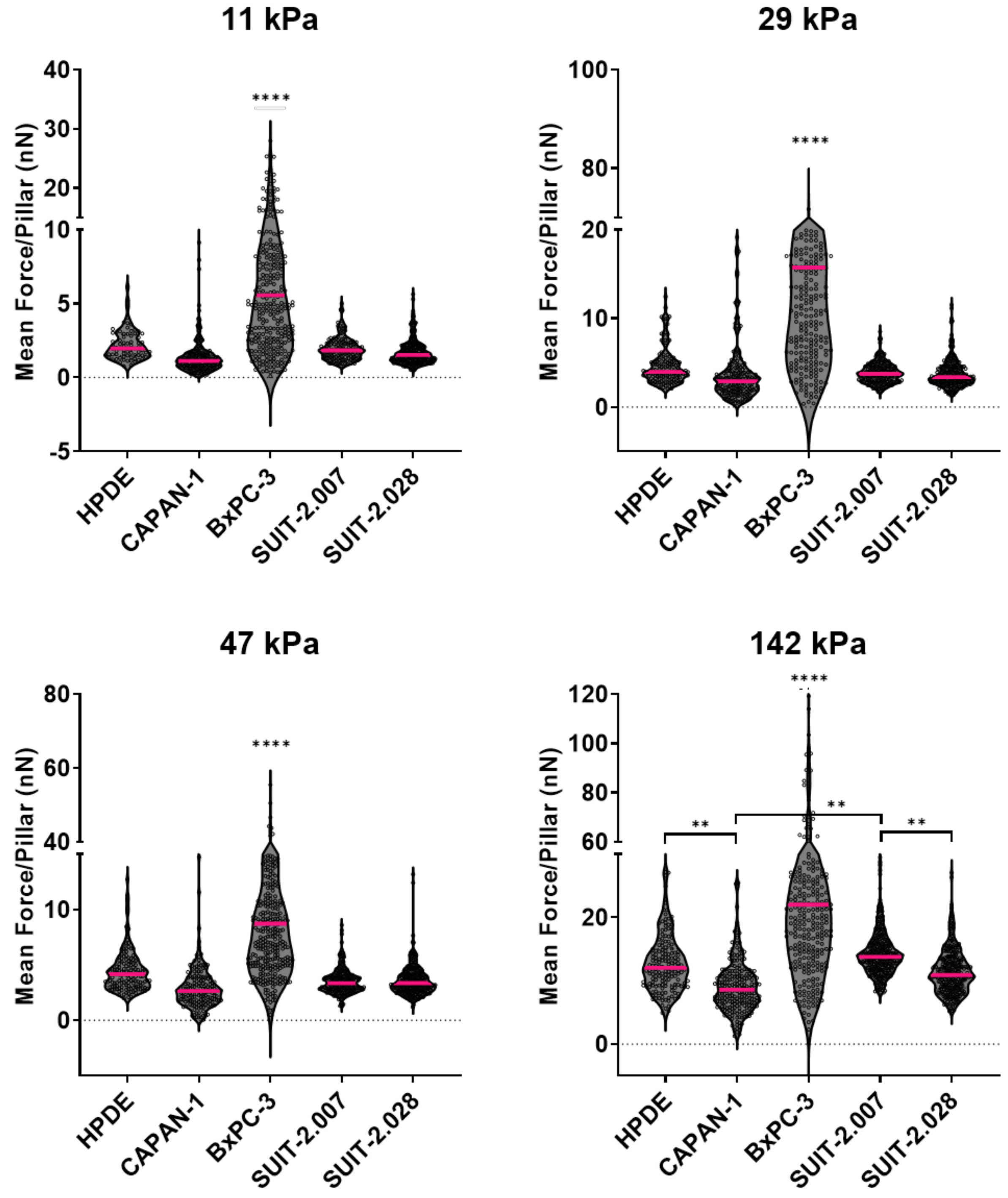

Supplemental Figure S3. Traction force of PDAC cells. Mean force per pillar (nN) of different PDAC cell lines growing on pillars with varying stiffness.

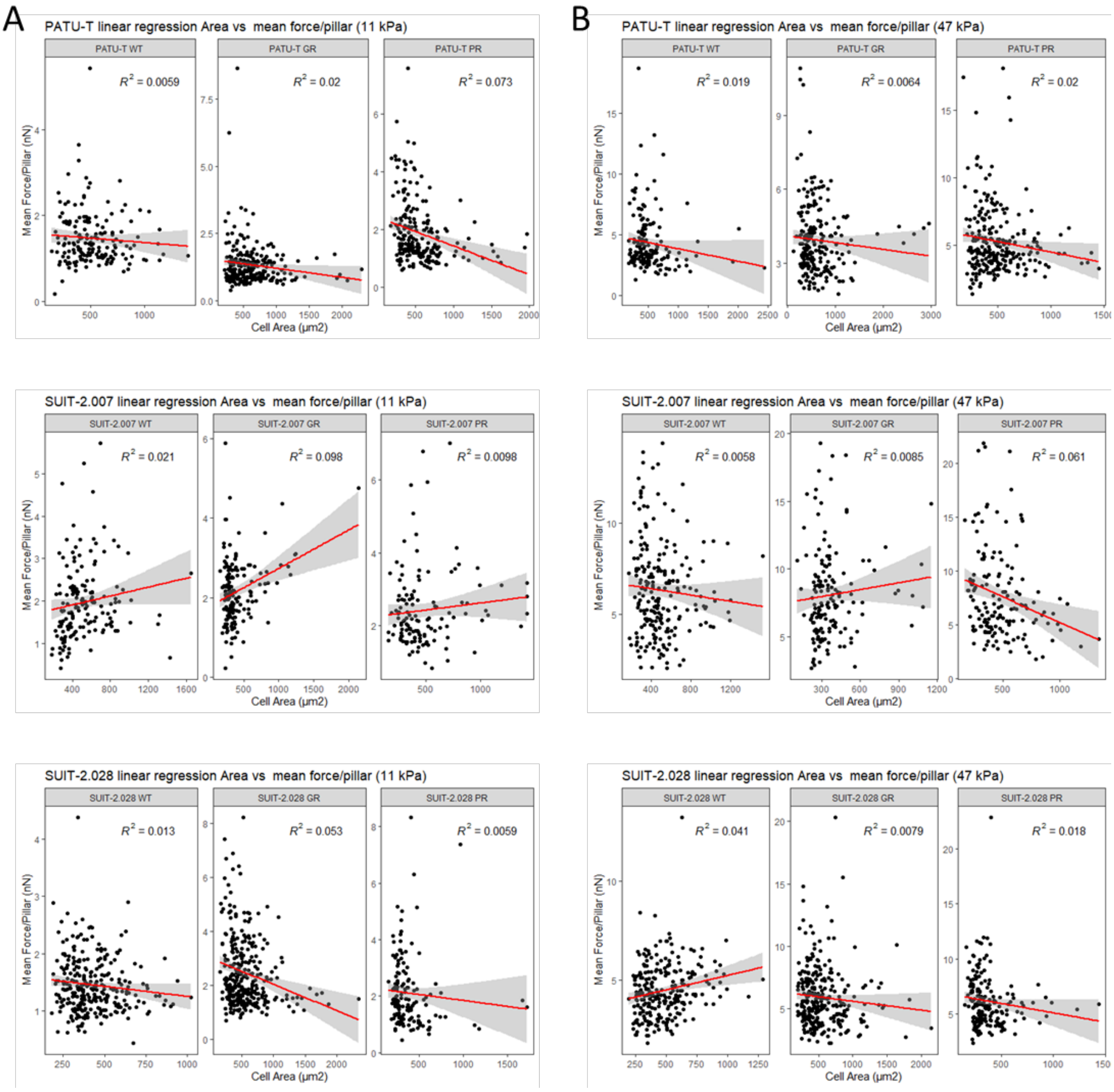

Supplemental Figure S4. Linear regression model of the mean force per pillar (nN) vs. spreading area (µm^2) of PDAC chemoresistant cells grown on (**A**) soft (11 kPa), and (**B**) stiff (47 kPa) pillars. Note that in all cases $R^2 \leq 0{,}02$, indicating that also for PDAC chemoresistant cells the mean force per pillar is uncorrelated to spreading area.

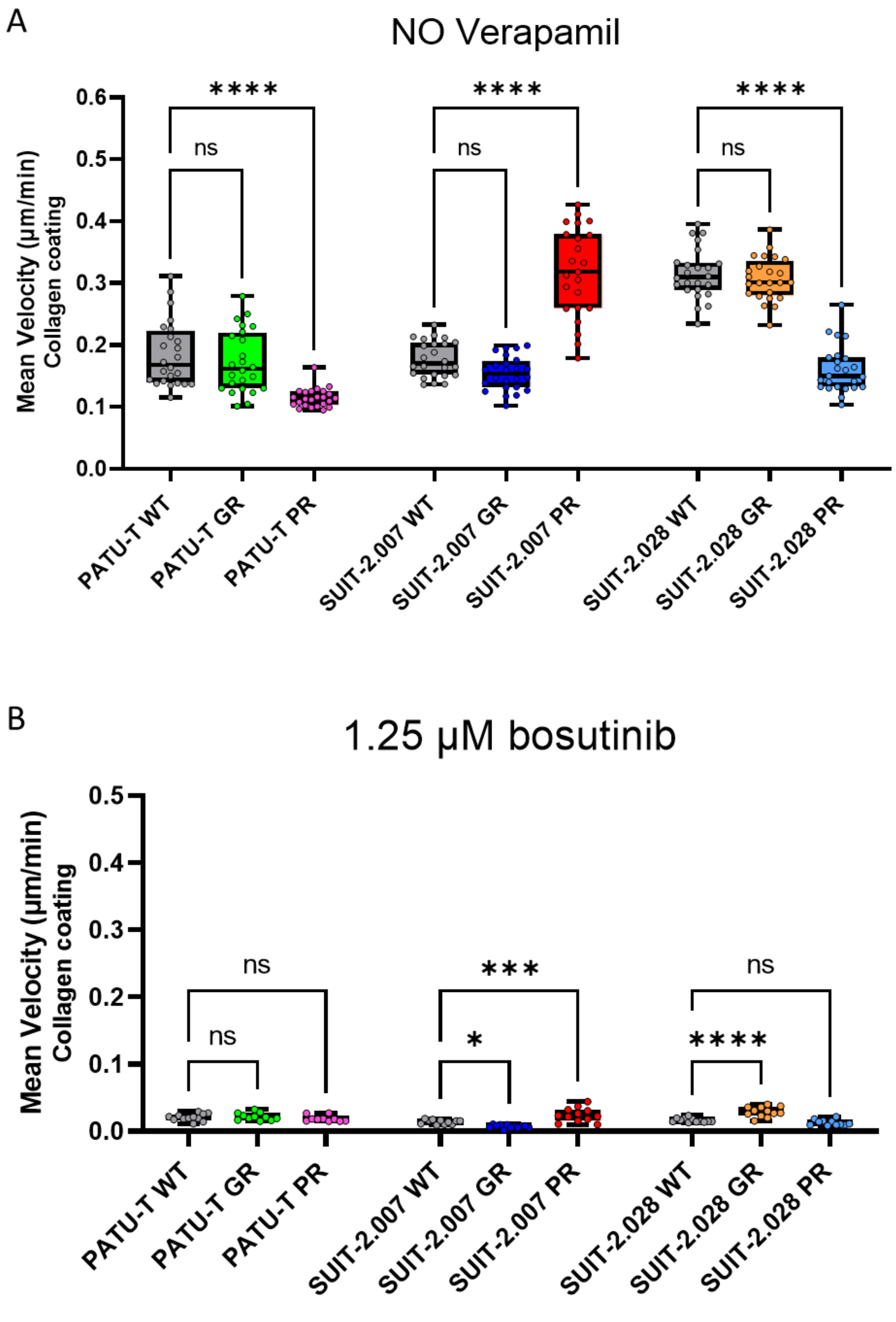

Supplemental Figure S5. PDAC cell migration is effectively inhibited by the motility-inhibitor bosutinib but not affected by the ABCB1-blocker verapamil. PDAC cell velocity, expressed as mean velocity (µm/min), growing on collagen-coated wells (**A**) untreated (no verapamil), and (**B**) treated with bosutinib (positive control).

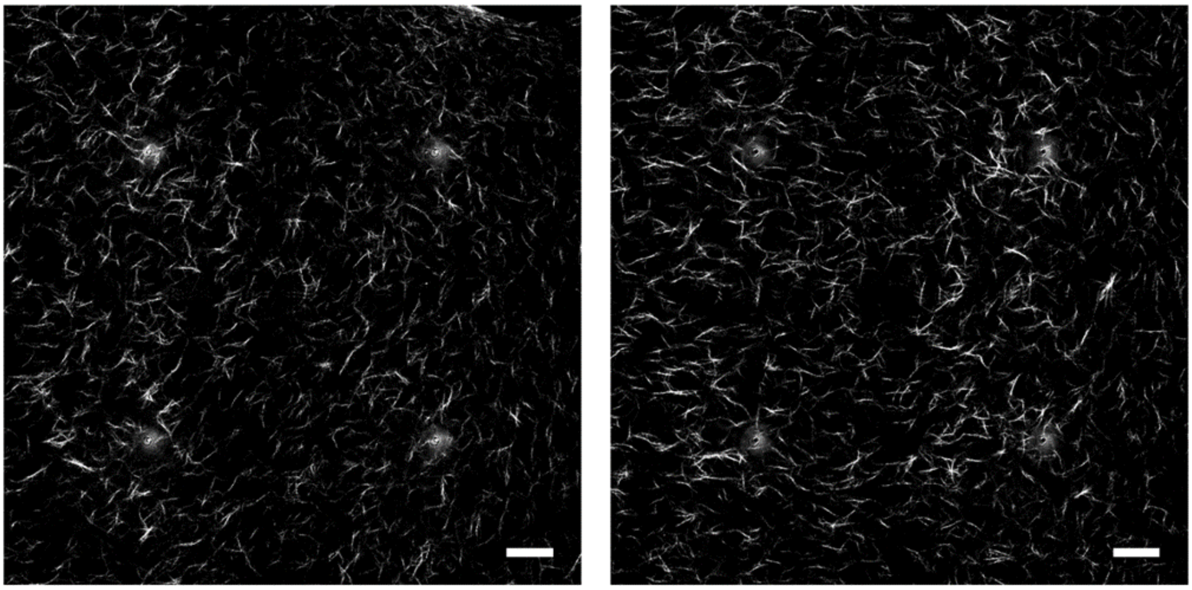

Supplemental Figure S6. Representative confocal reflection images of single z-planes of empty collagen gels, showing the random orientation of collagen fibers. Scale bar is 200 μm.

References

1. Schmidt, T.; Schütz, G.J.; Baumgartner, W.; Gruber, H.J.; Schindler, H. Imaging of Single Molecule Diffusion. *Proc. Natl. Acad. Sci.* **1996**, *93*, 2926–2929, doi:10.1073/pnas.93.7.2926.

2. Kusumi, A.; Sako, Y.; Yamamoto, M. Confined Lateral Diffusion of Membrane Receptors as Studied by Single Particle Tracking (Nanovid Microscopy). Effects of Calcium-Induced Differentiation in Cultured Epithelial Cells. *Biophys. J.* **1993**, *65*, 2021–2040, doi:10.1016/S0006-3495(93)81253-0.

3. Bobroff, N. Position Measurement with a Resolution and Noise-Limited Instrument. *Rev. Sci. Instrum.* **1986**, *57*, 1152–1157, doi:10.1063/1.1138619.
